# Electrochemical Sensor Based on Prussian Blue Electrochemically Deposited at ZrO_2_ Doped Carbon Nanotubes Glassy Carbon Modified Electrode

**DOI:** 10.3390/nano10071328

**Published:** 2020-07-07

**Authors:** Marlon Danny Jerez-Masaquiza, Lenys Fernández, Gema González, Marjorie Montero-Jiménez, Patricio J. Espinoza-Montero

**Affiliations:** 1 Escuela de Ciencias Químicas, Pontificia Universidad Católica del Ecuador, Quito 17 01 21-84, Ecuador; marlon.jerez@yachaytech.edu.ec (M.D.J.-M.); marjorie_cpp@hotmail.com (M.M.-J.); 2 School of Physical Sciences and Nanotechnology, Yachay Tech University, Urcuqui 100650, Ecuador; ggonzalez@yachaytech.edu.ec; 3 Departamento de Química, Universidad Simón Bolívar, Caracas 89000, Venezuela; 4 Instituto Venezolano de Investigaciones Científicas, Centro de Ingeniería Materiales y Nanotecnología, Caracas 89000, Venezuela

**Keywords:** carbon nanotubes, zirconia nanoparticles, Prussian blue, electrochemical sensors

## Abstract

In this work, a new hydrogen peroxide (H_2_O_2_) electrochemical sensor was fabricated. Prussian blue (PB) was electrodeposited on a glassy carbon (GC) electrode modified with zirconia doped functionalized carbon nanotubes (ZrO_2_-fCNTs), (PB/ZrO_2_-fCNTs/GC). The morphology and structure of the nanostructured system were characterized by scanning and transmission electron microscopy (TEM), atomic force microscopy (AFM), specific surface area, X-ray diffraction (XRD), thermogravimetric analysis (TGA), Raman and Fourier transform infrared (FTIR) spectroscopy. The electrochemical properties were studied by cyclic voltammetry (CV) and chronoamperometry (CA). Zirconia nanocrystallites (6.6 ± 1.8 nm) with cubic crystal structure were directly synthesized on the fCNTs walls, obtaining a well dispersed distribution with a high surface area. The experimental results indicate that the ZrO_2_-fCNTs nanostructured system exhibits good electrochemical properties and could be tunable by enhancing the modification conditions and method of synthesis. The fabricated sensor could be used to efficiently detect H_2_O_2_, presenting a good linear relationship between the H_2_O_2_ concentration and the peak current, with quantification limit (LQ) of the 10.91 μmol·L^−1^ and detection limit (LD) of 3.5913 μmol·L^−1^.

## 1. Introduction

A sensor is a device with the ability to transform a physicochemical process into an analytical signal. A sensor is made up of a recognition element and a transducer. The recognition element interacts with the analyte and the transducer, converting physicochemical changes into an electrical signal. Macrostructure systems-based sensors have been widely studied in order to explore their potential applications in clinical diagnostics [1]. Furthermore, the data given by sensors is needed to be accurate and reliable, thus, electrochemistry and analytic chemistry combined with nanotechnology are promising areas of investigation—in order to give the instrumentation precise methodology and accurate diagnostic capabilities. Electrochemistry provides analytical techniques, which are widely used due to its rapid response, low cost, portability and its facility to incorporate into complex researches. Electrochemical sensors are based on these techniques taking advantage of the redox processes of substances. The demand for sensors for the detection of harmful biological agents has increased, and recent researches are focused on ways of producing small portable devices that would allow fast, accurate, and on site detection [1,2]. In the case of biosensors, the recognition system is a biological element such as enzymes, antibodies, microorganisms, etc. Enzymes are substances which catalyze a chemical reaction with high selectivity, for instance—glucose oxidase will catalyze only the reaction of glucose. Oxidase enzymes are used in most of the investigations as the recognition element. Unfortunately, enzyme based biosensors suffer denaturation of the enzyme and different efforts have been done to improve their stability. Inorganic compounds that mimic the electrocatalytic activity of enzymes have been extensively studied in recent years [3,4,5,6,7,8,9].

Prussian blue (PB) is part of an important group of inorganic compounds used in electrode modification and for electrocatalytic purposes [10,11]. PB is known as an artificial peroxidase [12,13], and it is a promising candidate for the catalysis of H_2_O_2_. The use of PB as a recognition element for H_2_O_2_ detection is extensive [14,15,16]. Furthermore, in order to increase the sensitivity and the rapid response of electrochemical sensors, nanomaterials and nanostructured systems are used to modify the electrode surface, since it has been reported that nanomaterials promote electron transfer more efficiently between the electrodes [6,7,8,11,17,18,19]. On the other hand, carbon nanotubes possess interesting chemical and physical properties, which are useful in order to modify electrochemical sensors, therefore, the combination of PB, carbon nanotubes (CNTs), and metals have received special attention. Carbon nanotubes have good electrical conductivity; therefore, they can be used as a mediator for PB-modified electrodes. Electrochemical sensors for H_2_O_2_ detection are developed by adding electroactive materials (metals, metal oxides, enzymes, etc.) on the surface of electrodes. Hydrogen peroxide is a mediator found in the majority of biological reactions. You et al. [20] reported the electrocatalytic reduction of H_2_O_2_ using a system of Pd on CNTs. The electrode showed good linear range detection attributed to the improved sensitivity and selectivity. Wang, et al. [21] reported the determination of H_2_O_2_ on an electrode based on PB and CNTs. On the other hand, the use of zirconia nanoparticles involves applications such as: oxygen sensors, solid oxide fuel cells, H_2_ gas storage materials, catalysts and catalyst support, biosensors [22]. Nanostructured zirconia has been used as a biosensing platform for oral cancer detection using cyclic voltammetry techniques [23]. Additionally, zirconia nanoparticles grafted to collagen were used as an unmediated biosensing of H_2_O_2_, accelerating the electron transfer with good thermal stability [24]. Zirconia/multi-walled carbon nanotube nanocomposite was used to immobilize myoglobin showing excellent electrocatalytic activity to the H_2_O_2_ reduction [25]. In addition, zirconia/reduced graphene has been used as a high performance electrochemical sensing and biosensing platform [26], obtaining glucose oxidase immobilization successfully.

Considering the potential of CNTs and zirconia nanoparticles as biosensing platforms, in the present work a new H_2_O_2_ electrochemical sensor is proposed. PB is electrodeposited at a glassy carbon (GC) electrode modified with PB and zirconia doped carbon nanotubes.

## 2. Materials and Methods

The layer-by-layer method was used to deposit CNTs samples and poly (diallyldimethylammonium chloride) (PDDA), and PB film were deposited electrochemically at the GC electrode. PDDA is a conductor polymer positively charged [27], this film will provide an electrostatic interaction between the CNTs negatively charged and GC electrode. By using cyclic voltammetry (CV), the electron transfer behavior and electrocatalysis of PB were investigated. Additionally, cyclic voltammetry response and chronoamperometry response of the modified electrodes in the presence and absence of H_2_O_2_ were studied.

### 2.1. Materials and Reagents

All solutions were prepared with distilled/deionized water (18 MΩ resistivity, Darmstadt, Germany). Carbon nanotubes were obtained from Material Science & Nanotechnology Laboratory (IVIC, Caracas, Venezuela). Nitric acid (HNO_3_, 69.2 wt.%) and hydrogen peroxide (H_2_O_2_, 30%V/V) were purchased from Sigma-Aldrich Sigma, (Darmstadt, Germany). Potassium phosphate monobasic (KH_2_PO_4_) and sodium hydroxide (NaOH, 99.9% p/p) were purchased from Fisher Scientific (Waltham, MA, USA). Phosphate-buffered saline (PBS, 20 mmol·L^−1^ KH_2_PO_4_ + 20 mmol·L^−1^ K_2_HPO_4_ + 0.1 mol·L^−1^ KCl, pH 6.8) was used as a supporting electrolyte. Potassium ferricyanide (K_3_[Fe(CN)_6_]), iron trichloride hexahydrated (FeCl_3_·6H_2_O), and potassium chloride (KCl) were from BDH Chemicals (Philadelphia, PA, USA), hydrochloric acid (HCl, 37%) was from Fisher Scientific (Waltham, MA, USA), glassy carbon (GC, diameter (Φ=3 mm), geometric area = 0.0706 cm^2^), silver/silver chloride reference electrode (Ag/AgCl), and graphite rod counter-electrode were from CH-Instruments (Austin, TX, USA), sulfuric acid (H_2_SO_4_, 98%) was from Fisher Scientific (Waltham, MA, USA), and 1 µm, 0.3 µm, and 0.05 µm alumina powder were from CH-Instruments (Austin, TX, USA), dimethylformamide (DMF) was from BDH Chemicals (Philadelphia, PA, USA), poly (diallyldimethylammonium chloride) (PDDA, 4% w/w in water) were from Sigma.

### 2.2. Carbon Nanotubes (CNTs) Functionalization

The functionalization of carbon nanotube and nanostructured materials was carried out in a previous work [28]. The functionalization was made in two parts the pre-functionalization and functionalization:

### 2.3. Pre-Functionalization of CNTs

First, 1.0 g of CNTs were added in a 500 mL volumetric flask, and then 100 mL of 3.0 mol·L^−1^ of nitric acid and 300 mL of 1.0 mol·L^−1^ of sulfuric acid were added in this order. The reaction mixture was placed under a reflux at a temperature of about 80 °C and a stirring speed of 400 rpm for six hours. Once the mixture was cooled at room temperature, CNTs were filtered in a frit of porous plate, and washed with deionized water until the wash solution reach a neutral pH. Afterward, the washed CNTs were dried in an oven under low vacuum at 84.42 kPa and 60 °C for 12 h, finally CNTs were ground in a mortar.

### 2.4. Functionalization of CNTs

To add carboxylic (−COOH) and hydroxyl (−OH) groups to the side-wall of CNTs the following procedures were performed. Then, 80 mL solution of nitric acid 60 wt.% was added in a 250 mL volumetric flask with pre-functionalized CNTs. The mixture was placed under ultrasonic agitation for 30 min and then under reflux at 80 °C and 400 rpm for two hours. Then, the mixture was cooled at room temperature and diluted with 200 mL of deionized water, next, the filtering process was made as described in pre-functionalization section. Subsequently, CNTs were dried in an oven under low vacuum at 84.42 kPa and 60 °C for 16 h, finally CNTs were ground in a mortar and then sieved using a 125 µm sieve.

### 2.5. Synthesis of ZrO_2_-fCNT Nanostructured System

The ZrO_2_ was synthesized in situ on the functionalized CNTs (fCNTs). The synthesis was described in a previous work [28]. Half volume of propanol was added to a volumetric flask with fCNTs, and then it was subjected to ultrasonic agitation. Then, zirconia isopropoxide (Zr(OPri)_4_), 1/4 volume of isopropanol, and acetic acid were added to other volumetric flask, and this mixture was subjected to ultrasonic agitation for 10 min. Afterward, the above mixture as placed in an addition vessel to drop it on the isopropanol and fCNTs solution, which was subjected to mechanical agitation at 600 rpm at room temperature. Next, isopropanol in deionized water solution was added drop by drop to the previous mixture. After adding the reagents, the reaction was maintained for two hours, and allowed to age 20 days. After aging time, the mixture was placed in a beaker, washing the volumetric flask with alcohol. The solvent was evaporated at about 88 °C until a pasty mixture was obtained. Then, deionized water, three times of volume of mixture, were added to the pasty mixture, which were evaporated at temperatures between 88–96 °C. Subsequently, the resulting substance was dried in low vacuum at 80 °C for 4 h, afterward; a thermal treatment was performed in argon atmosphere at 50 °C for 2 h. Finally, the sample was grounded in a mortar.

### 2.6. Material Characterization

Transmission electron microscopy (TEM) images were taken in a JEOL 1220 microscope (Jeol, Peabody, MA, USA) at acceleration voltages of 100 and 200 kV. Scanning electron microscopy (SEM) analysis was carried out in a field emission scanning electron microscopy (FESEM) Hitachi F2400 (Hitachi, Krefeld, Germany), operating at 30 kV and 10 kV attached with an energy dispersive spectrometer (EDS) Quantax75. Analysis by atomic force microscopy (AFM, Agilent, Santa Clara, CA, USA) in tapping mode was made in an Agilent 5500 equipment. A long cantilever point probe-plus (L = 225 ± 10 µm) with a force constant of 21–98 N m^−1^, and a silicon tip was used at a resonance frequency of 156.42 kHz. Topographic, amplitude and phase images were recorded using a scanning speed of 4 l s^−1^. Areas of 2.5 × 2.5 and 5.0 × 5.0 µm were scanned using a high-resolution multipurpose scanner. Samples for TEM and AFM were prepared by adding a tiny amount of sample to a solution of ethanol/water. This suspension was sonicated for 10 minutes and a drop of this suspension was placed in a TEM holey carbon grid for TEM analysis, and on a (100) silicon wafer (atomic roughness) for AFM analysis. The specific surface area was determined via Brunauer–Emmett–Teller (BET) method using a Micromeritics ASAP2010 equipment (Micromeritics, Norcross (Atlanta), GA, USA). The adsorption-desorption isotherms were measured at 77 K with liquid nitrogen, previously degassing of the samples at 150 °C, for 4 h under vacuum. Raman spectroscopy was performed in a high-resolution confocal Raman spectrometer Dilor XY800 (Horiba, Kyoto, Japan) operating in backscatter mode with a high definition CCD detector, cooled at 77 K with liquid nitrogen. An argon laser with a wavelength of 514.5 nm. X-ray diffraction (XRD) spectrum of samples was registered by SIEMENS D5005 diffractometer (SIEMENS, Malvern, United Kingdom), at a wavelength (λ) of 1.541 Å. The samples were analyzed in the range of 2θ = 10°–80° at a scan rate of 0.02°/0.52 s. Fourier transform infrared (FTIR) spectrum were recorded in a Nicolet iS10 FTIR spectrometer (Thermo Fisher Scientific, Waltham, MA, USA), with a resolution of 4 cm^−1^. Samples in powder form were evaluated in KBr pellets with a concentration of 0.2 to 1% in the pellet. Zeta potential measurements were done in an aqueous suspension of carbon nanotubes samples with distilled water as the solvent at a temperature of 24.1 °C and humidity of 45.7%. Both samples were submitted to 5 runs of measure. Thermal analysis DSC-TGA of the pristine, functionalized and ZrO_2_ doped fCNTs, under air atmosphere, using a TA Instrument SD Q600 (TA Instrument, New Castle, DE, USA), at a heating rate of 10 °C/min, from room temperature to 1000 °C.

### 2.7. Electrode Modification

fCNTs and zirconia doped fCNTs (ZrO_2_-fCNT) were suspended in DMF at a concentration of 5.0 mg/mL, then this suspension was sonicated about 15 min, and storage at room temperature and sealed with parafilm. A volume of 10 µL of fCNTs suspension was pipetted on the surface of the GC electrode. The GC electrode was allowed to dry during 15 min at 50 °C, after that 10 µL of the PDDA solution was pipetted on the modified GC electrode and dry during 15 min at 50 °C. Once the GC electrode was modified with fCNTs and ZrO_2_-fCNTs, the PB was electrodeposited.

**Scheme 1:**
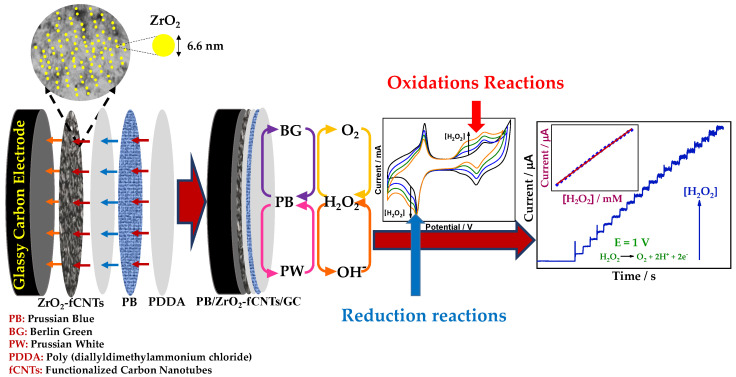
Modification of PB/ZrO_2_-fCNT/GC electrode and mechanism of H_2_O_2_ detection.

Electrodeposition of PB was accomplished in an aqueous solution containing 2.5 mmol·L^−1^ K_3_[Fe(CN)_6_] + 2.5 mmol·L^−1^ FeCl_3_·6H_2_O + 0.1 mol·L^−1^ KCl + 0.1 mol·L^−1^ HCl at potential of +0.4 V during 60 s. Subsequently, the PB-electrodeposited electrode was activated by cycling at a potential range from −0.2 V to 1.2 V at scan rate of 50 mV·s^−1^ in 0.1 mol·L^−1^ KCl + 0.1 mol·L^−1^ HCl solution for 20 cycles. The PB based electrode was allowed to dry at 50 °C for 15 min, afterward, a volume of 10 µL of PDDA solution was cast on the modified electrode and dried during 15 min at 50 °C. Finally, the PB/fCNT/GC electrode was obtained. The PB/ZrO_2_-fCNT/GC electrode was prepared also by the procedure describe above (Scheme 1). The modified GC electrodes were rinsed twice with distilled water and stored at room temperature.

### 2.8. Electrochemical Characterization

The electrochemical experiments were performed on a CHI604A (CH Instruments, Austin, TX, USA) electrochemical workstation with a conventional three-electrode system. The GC modified electrodes were used as the working electrodes, a graphite rod as the counter electrode and Ag/AgCl (saturated KCl) as the reference electrode. The supporting electrolyte used was the 0.1 mol·L^−1^ PBS solution (pH 6.8). The stability of the electrode was reach by cycling from 10 to 100 times.

### 2.9. H_2_O_2_ Detection

The response of modified electrodes in the presence of H_2_O_2_ were tested by chronoamperometry (CA) and cyclic voltammetry (CV). CA experiments were carried out by applying a constant potential of 1.0 V vs. Ag/AgCl (saturated KCl) in a stirred 0.1 mol·L^−1^ PBS (pH 6.8). Aliquots of H_2_O_2_ were added successively every 20 s. CV measurements were performed at a potential range from −0.2 V to 1.2 V at a scan rate of 40 mV·s^−1^ for one cycle. H_2_O_2_ detection was performed at temperatures lower than 10 °C.

## 3. Results and Discussion

### 3.1. Fourier Transform Infrared (FTIR) Spectroscopy

**Figure 1:**
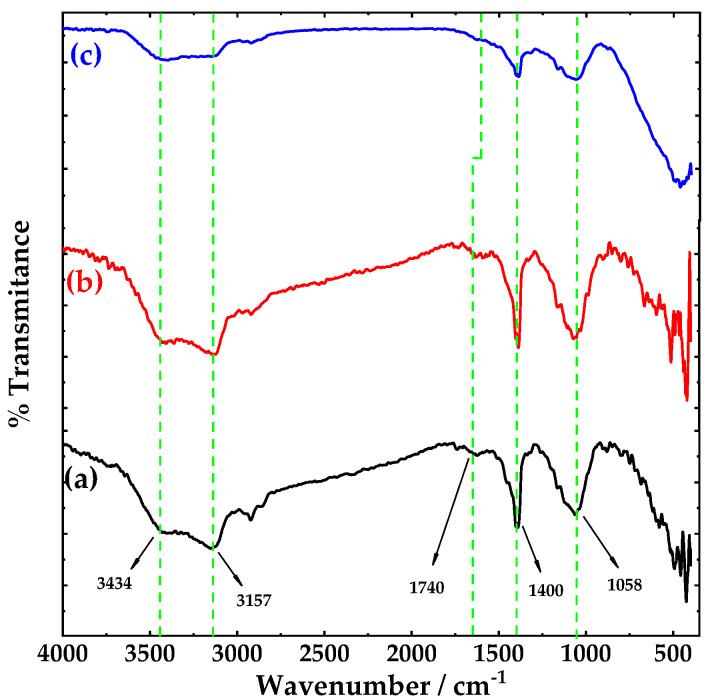
FTIR spectra of (**a**) pristine carbon nanotubes (CNTs), (**b**) functionalized CNTs and (**c**) ZrO_2_-fCNTs in a range from 4000–400 cm^−1^.

**Figure 2:**
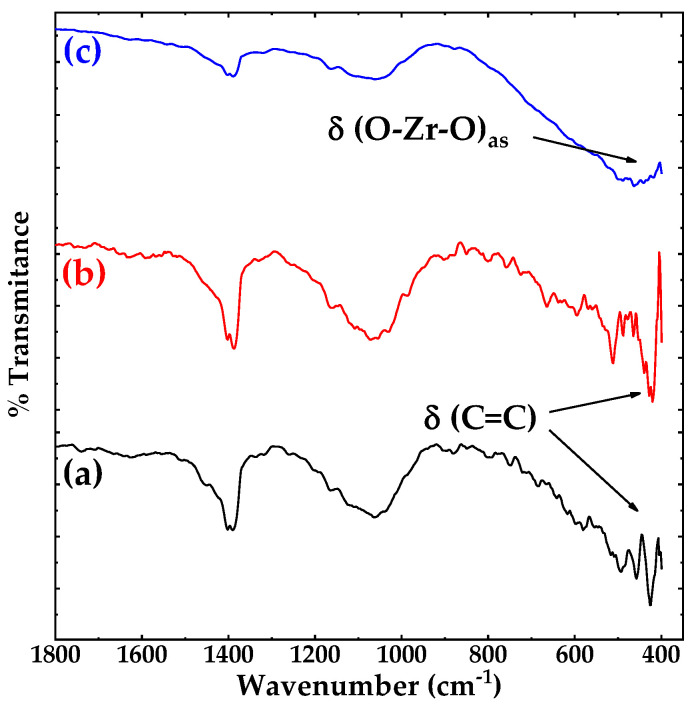
FTIR spectra of (**a**) pristine CNTs, (**b**) functionalized CNTs and (**c**) ZrO_2_-fCNTs in a range from 1800–400 cm^−1^.

Figure 1 shows the FTIR spectra of: (a) pristine carbon nanotubes (pCNTs), (b) functionalized CNTs (fCNTs) and (c) zirconia doped carbon nanotubes (ZrO_2_-fCNTs) nanostructured system. FTIR spectra shows similar spectra for CNT and fCNTs samples, this can be attributed to the weak charge difference between the carbon atoms due to the high symmetry of CNTs which generates weak signals in the infrared spectrum [29]. Furthermore, the pristine, functionalized and ZrO_2_ doped CNTs show vibration bands at 1400 cm^−1^, attributed to vibration modes of multi-walled carbon nanotubes (MWCNTs) [30], and a very weak band at 1740 cm^−1^ assigned to C=O vibration of the carboxylic groups, present from the purification and functionalization treatments. Titration experiments to measure the concentration of functional groups on the CNTs walls resulted in 3 × 10^−3^ mol·g^−1^. The broad band around 4300 cm^−1^ and the band at 1630 cm^−1^ correspond to hydroxyl groups. The binding of ZrO_2_ on the MWCNT sidewalls surface is associated to the small shift observed in the C=O band of the carboxylic group (Figure 1c). Moreover, it has been reported that carbon groups stabilize the growth of zirconia nanoparticles on the fCNTs surface [31]. The C=C, C–H and C–O–C vibration bands are in the region of 1200 to 1550 cm^−1^ inherent to CNTs structure. Figure 2 presents the fingerprint region of CNTs (a) and fCNTs (b) in a range of 400 to 1800 cm^−1^. Figure 2c shows a broad band between 400–750 cm^−1^ corresponding to ZrO_2_ vibration modes with a broad maximum at 480 cm^−1^ assigned to Zr–O–Zr asymmetric stretching and deformation modes [32].

### 3.2. Raman Spectroscopy

**Figure 3:**
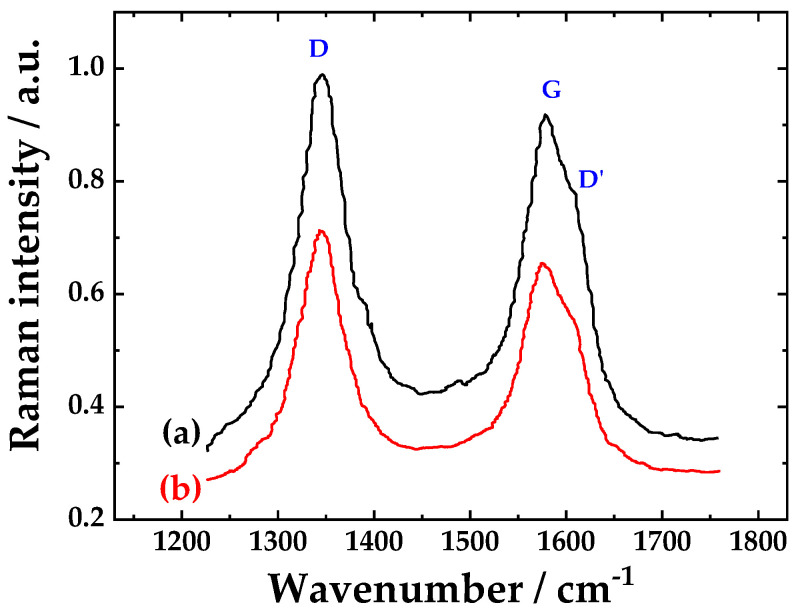
Raman spectra of (**a**) pristine CNTs and (**b**) functionalized CNTs.

The Raman spectra of the pristine and functionalized CNTs are shown in Figure 3. Three characteristic bands can be observed at ≈1340 cm^−1^, the D-band known as the disorder mode due to the graphitic planes and other forms of carbon and defects present on the nanotube walls. At ≈ 1570 cm^−1^ the G-band graphite mode, corresponding to planar vibration of carbon atoms in graphene and a weak shoulder of the G-band, at higher frequencies ≈1610 cm^−1^, D′ is present as a double resonance feature induced by disorder and defects [33].

The I_D_/I_G_ ratio is an indication of the quality of the sample [33]. The I_D_/I_G_ ratio for the pristine sample was 1.06. This ratio increases in the fCNTs to 1.17 due to addition of new defect sites on the walls from the oxidizing treatment.

### 3.3. Surface Area

**Figure 4:**
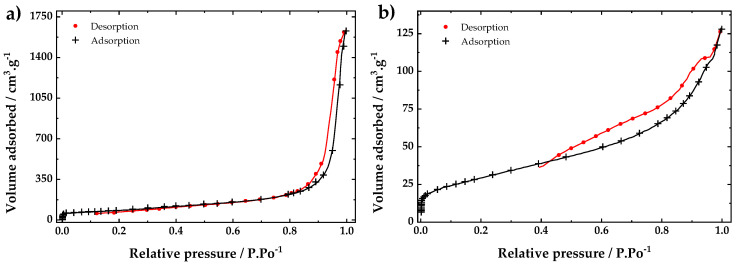
Adsorption-desorption isotherms of (**a**) fCNTs and (**b**) ZrO_2_-fCNTs.

The adsorption-desorption isotherms of fCNTs and ZrO_2_-fCNTs are presented in Figure 4. The specific surface area given by Brunauer–Emmett–Teller (BET), and pore volume by Barrett–Joyner–Halenda (BJH), and are summarized in Table 1. The adsorption-desorption isotherm for the modified ZrO_2_-fCNTs is type IV, according to International Union of Pure and Applied Chemistry (IUPAC) [34]. The hysteresis observed indicates the formation of mesopores in the hybrid material [34]. The specific surface area and pore volume decrease for the ZrO_2_-fCNTs, due to the decoration of the nanoparticles on the nanotubes walls. Therefore, the attachment of ZrO_2_ affects the surface area, pore volume and pore size distribution.

### 3.4. Thermal Analysis

The thermodegradation process of pristine (pCNTs) and functionalized (fCNTs) samples shows that the decomposition process occurred in one stage with Ti 426 °C and 504 °C, respectively, Table 2. The lower Ti of pCNTs is probably due to the presence of amorphous carbon present at the surface, but eliminated from the fCNTs during the oxidative treatment. The total decomposition of fCNTs is higher since they have a smaller number of walls and fewer impurities. The ZrO_2_-fCNT presents a first weight loss at low temperatures 80–150 °C, attributed to desorbed water, then a two stage decomposition, the first between 276–347 °C with a weight loss of 2% attributed to decomposition of physiosorbed organic species, from remaining residues of zirconium isopropoxyde compound used in the synthesis of the zirconia nanoparticle, and a second stage of CNTs decomposition between 366–597 °C with 40% weight loss, Table 2. The effect of ZrO_2_ nanoparticles lowering the decomposition temperature of fCNTs is well known, with a catalyzing effect on carbon decomposition. The percentage of ZrO_2_ present in the nanocomposite was 40%, in good agreement with the initial amount added during the synthesis.

### 3.5. Zeta Potential

**Figure 5:**
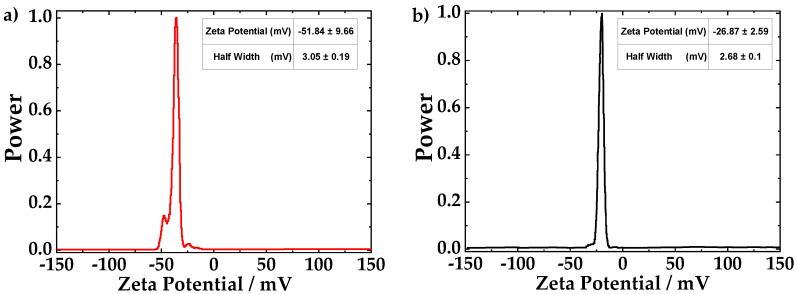
The zeta potential curve of (**a**) fCNTs and (**b**) ZrO_2_-fCNTs in an aqueous solution.

The zeta potential technique was used to understand the electrostatic interactions in the hybrid CNTs dispersions. An electrical field is applied across the suspensions which vary the movement of the particles [35]. The mobility of particles gives information of the charge present. Furthermore, the colloidal stability is controlled by the electrostatic interactions of the particles and the solvent [36]. The zeta potential spectrum of fCNTs is shown in Figure 5a. The mean value of zeta potential is −51.84 mV with a standard deviation of 9.66 mV. The negative charge of fCNTs is due to the deprotonation of functional groups (carboxylic and hydroxyl groups) which were attached to the CNTs walls during the acidic treatment. Figure 5b shows the zeta potential measurement of the ZrO_2_-fCNTs sample with a mean value of −26.87 mV and a standard deviation of 2.69 mV. The ZrO_2_ nanoparticles are attached to the functional groups, which neutralize part of the fCNTs charge, then the zeta potential of ZrO_2_-fCNTs is lower than for fCNTs. Clogston and Patri [35], reported that the nanoparticles with zeta potential values between ±10 mV are considered neutral while the values greater than +30 mV or less than −30 mV are considered strongly cationic or anionic, respectively. Therefore, the solutions of fCNTs, and ZrO_2_-fCNTs can be considered strongly anionic and moderated anionic, respectively. The charge of the CNTs plays an important role in the colloidal stability and the dispersion of CNTs on the electrode surface. As the charge of CNTs are anionic, the solvent of both samples will be the same. DMF was the solvent selected to suspend both CNTs samples. Both suspensions, fCNTs and ZrO_2_-fCNTs, presented high stability.

### 3.6. X-Ray Diffraction (XRD) Spectroscopy

**Figure 6:**
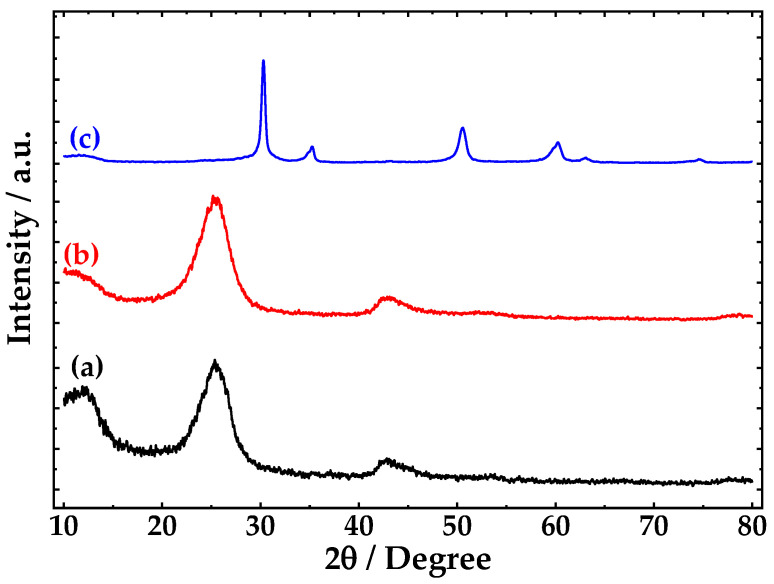
X-ray diffraction (XRD) pattern of (**a**) pristine CNTs, (**b**) functionalized CNTs and (**c**) ZrO_2_-fCNTs.

Figure 6, shows the diffraction pattern of CNTs, fCNTs and ZrO_2_-fCNTs nanostructured system. The XRD pattern of CNTs (Figure 6a,b) are similar to those of graphite, therefore the peaks at about 25° and 43° correspond to (002) and (100) of the honeycomb lattice of single graphene sheet [37]. XRD patterns do not show other reflections, which means the lower presence of carbonaceous impurities or other kind of impurities (i.e., metal oxides). With the functionalization process, the diffraction angle of (002) plane decreases while the interplanar distance and intensity increases, as it is shown in Table 3. This is related to the decrease of the outer CNT diameter [38].

XRD pattern of ZrO_2_-fCNTs (Figure 6c) shows the zirconia reflections. The 2θ values of ZrO_2_-fCNTs pattern match very well with the values of the cubic phase of ZrO_2_ reported by Luo, T. Y. et al. [39]. (Table 4). The presence of CNTs reflections is not evident due to the amount of CNTs (36%) respect to ZrO_2_ used. Using the Scherrer’s equation [40], the crystal size of ZrO_2_ obtained was 8.1 nm.

Zirconia has three crystalline phases: cubic stable at temperatures greater than 2370 °C, whereas the tetragonal is stable in the temperature range from 1170 to 2370 °C and monoclinic phase stable at temperatures lower than 1170 °C. To obtain stabilized high temperature phases at room temperature, zirconia has to be stabilized with yttrium or other dopants. However, for small crystal size it has been found that zirconia the high temperature phases are stable at room temperature depending on crystal size [41,42]. The presence of CNTs stabilizes the ZrO_2_ cubic phase [43]. The cubic phase has high ionic conductivity and low thermal conductivity, and in small crystal, size has a good electrical conductivity, therefore it has been used as an oxygen sensors and solid oxide fuel cells, due to the ability of oxygen ion to move freely through the crystal structure [44].

### 3.7. Scanning Electron Microscopy (SEM), Transmission Electron Microscopy (TEM) and Atomic Force Microscopy (AFM)

**Figure 7:**
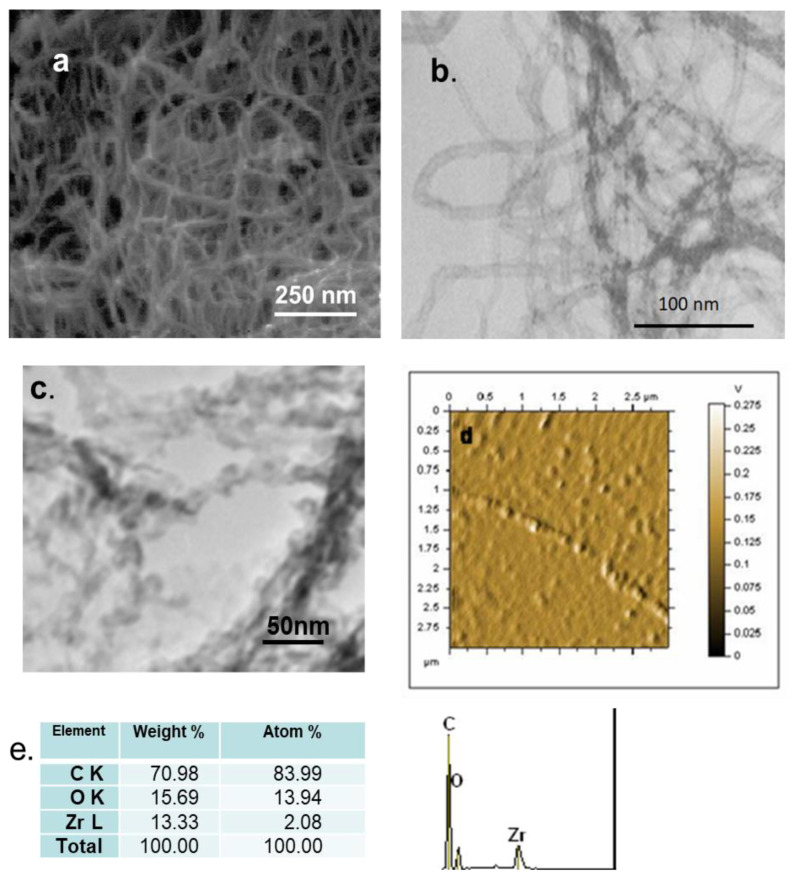
Morphological characterization: (**a**) field emission scanning electron microscopy (FESEM) image of ZrO_2_-fCNTs. Transmission electron microscopy (TEM) image of (**b**) fCNTs and (**c**) ZrO_2_-fCNTs nanostructure system. (**d**) Atomic force microscopy (AFM) amplitude image of ZrO_2_-fCNTs. (**e**) Energy dispersive spectrometer (EDS) analysis of area in (**a**).

SEM, TEM and AFM analysis of the different materials are shown in Figure 7. FESEM image of ZrO_2_-fCNTs sample is shown in Figure 7a, zirconia nanoparticles can be observed decorating the carbon nanotubes. The EDS analysis showed the presence of zirconium, oxygen and carbon (Figure 7e). TEM images show the characteristic morphology of fCNTs with an average diameter 12 ± 2 nm (Figure 7b), the ZrO_2_-fCNTs image can be observed in Figure 7c, showing the rounded zirconia nanoparticles with a random distribution on the fCNTs walls. Table 5 shows that the diameter of CNTs has decreased after functionalization due to some degradation of nanotube’s walls during the acid treatment process. The particle size of zirconia nanoparticles is 6.6 ± 1.8 nm in good agreement with the size obtained by XRD. Figure 7d is the AFM image in amplitude mode of carbon nanotube decorated with zirconia nanoparticles.

### 3.8. Electrochemical Characterization

**Figure 8:**
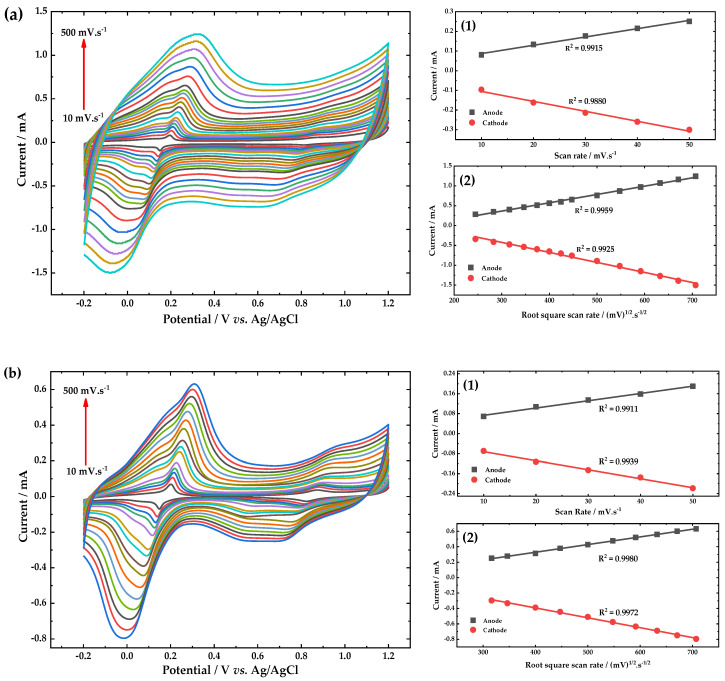
Cyclic voltammograms response of the (**a**) PB/fCNTs/GC and (**b**) PB/ZrO_2_-fCNTs/GC modified electrodes, in a 0.1 mol·L^−1^ PBS (pH 6.8) solution, at scan rates between 10–500 mV·s^−1^. Inset (**1**) current vs. scan rate, and inset (**2**) current vs. (scan rate)^1/2^.

Cyclic voltammograms of the PB/fCNTs/GC and PB/ZrO_2_-fCNTs/GC modified electrodes, in a 0.1 mol·L^−1^ PBS (pH 6.8) solution, were performed at scan rates between 10–500 mV·s^−1^ (Figure 8). The current signal between +0.1 and +0.3 V is associated to redox reactions of high spin $\mathrm{Fe}{\left(\mathrm{CN}\right)}_{6}^{3-/4-}$ (PB/PW redox reaction, reaction 1), and another current signal, between 0.7 V and 1.0 V, correspond to electrochemical reactions of low spin ${\mathrm{Fe}}^{3+/2+}$ (Prussian blue/Berlin green redox reaction, reaction 2) [45,46].

(R1) $${\mathrm{Fe}}_{4}^{\mathrm{III}}{\left[{\mathrm{Fe}}^{\mathrm{II}}{\left(\mathrm{CN}\right)}_{6}\right]}_{3}+4e^{-}+4K^{+}⇄K_{4}{\mathrm{Fe}}_{4}^{\mathrm{II}}{\left[{\mathrm{Fe}}^{\mathrm{II}}{\left(\mathrm{CN}\right)}_{6}\right]}_{3}$$

PB                 PW

(R2) $${\mathrm{Fe}}_{4}^{\mathrm{III}}{\left[{\mathrm{Fe}}^{\mathrm{II}}{\left(\mathrm{CN}\right)}_{6}\right]}_{3}+4A^{-}⇄K_{4}{\mathrm{Fe}}_{4}^{\mathrm{III}}{\left[{\mathrm{Fe}}^{\mathrm{III}}{\left(\mathrm{CN}\right)}_{6}A\right]}_{3}+3e^{-}$$

PB                 BG

where A^−^ is the anion supplied by the electrolyte.

For PB/fCNTs/GE modified electrode, in the reduction zone, the I_pa_/I_pc_ has an average of 0.86, which indicates an almost-complete reversibility at the electrode; while peak-to-peak separation potential (∆E_p_) is +0.26 V showing a higher resistance produced by the layers. In the oxidation zone the I_pa_/I_pc_ average is 1.13 and ∆E_p_ is +0.16 V [47]. The PB/ZrO_2_-fCNTs/GC modified electrode, in the reduction zone, displays a I_pa_/I_pc_ of 0.79 and the ∆E_p_ is +0.28 V; while in the oxidation zone, displays a I_pa_/I_pc_ of 1.6 and the ∆E_p_ is +0.27 V. These results, suggest a quasi-reversible reaction processes of the PB at the modified electrode surfaces. For both modified electrodes, the E_1/2_ was almost independent of the scan rate. The electrochemical results demonstrate that PB maintains good electrochemical activity at both modified electrodes [48].

Figure 8 shows that the peak current of PB in both composite films (fCNTs and ZrO_2_-fCNTs) increases linearly with scan rate up to 50 mV·s^−1^ (Figure 8a,b, Inset 1) indicating a surface-limited redox process. At scan rates higher than 50 mV·s^−1^, the plot of peak current of PB vs. $\nu^{1/2}$ in both composite films is linear, revealing a diffusion-controlled process (Figure 8a,b, Inset 2), according to the Randles–Sevcik equation, which we have related to the slow diffusion of potassium and/or sodium ions into the composites lattice. This indicates that the reaction kinetics changes with the scan rate interval [49].

The surface concentration ($\Gamma_{C}$) of PB in modified electrodes was calculated, using the cathodic peak located around 0.3 mV, from the slope I_p_ vs. ν (Figure 8a,b, Inset 1), according to Equation (1) [50], assuming a transfer of four electrons per unit cell of PB [51]:

(1) $$I_{p}=\frac{n^{2}F^{2}{A\nu \Gamma }_{C}}{4\mathrm{RT}}$$

The average value of $\Gamma_{C}$ for the redox peaks was 3.98 × 10^−10^ and 1.2×10^−9^ mol·cm^−2^ at PB/fCNTs/GC and PB/ZrO_2_-fCNTs/GC modified electrode (for ν < 500 mV s^−1^), respectively. Increasing the time of electrodeposition of the PB film on both modified electrodes from 100 up unaffected $\Gamma_{C}$ value. The deposition time was fixed to 240 s for further investigations because deposition at longer times produced CVs with higher capacitive currents and ΔEp values higher than 0.8 V. PB should be preferentially electrodeposited on the ZrO_2_-fCNTs/GC electrode, since the $\Gamma_{C}$ calculated value is the largest on the PB/ZrO_2_-fCNTs/GC electrode. These results are fully consistent with the idea of a PB-ZrO_2_ preferably interacting within the ZrO_2_-fCNTs composite host. One of the reasons may be that the molecular PB can enter the cavity of the ZrO_2_ and electrodeposited on the ZrO_2_ host. The BET surface area of fCNTs was 298.40 ± 2.72 m^2^·g^−1^ and for the nanostructured ZrO_2_-fCNTs was 92.12 ± 1.03 m^2^·g^−1^, however from the results it could be inferred that both CNTs and the ZrO_2_ nanoparticles are responsible for the direct electron transfer offering a synergistic effect. The carbon nanotubes accelerate the electron transfer providing conductive pathways and increase the conductivity of the matrix and the zirconia nanoparticles offering a more favorable surface for PB electrodeposition. This is probably related to the number of ZrO_2_ particles attached to the nanotube’s wall and their particle size, which according to Du et al. [52] and Karyakin et al. [53], this can be the cause of a series of effects.

**Figure 9:**
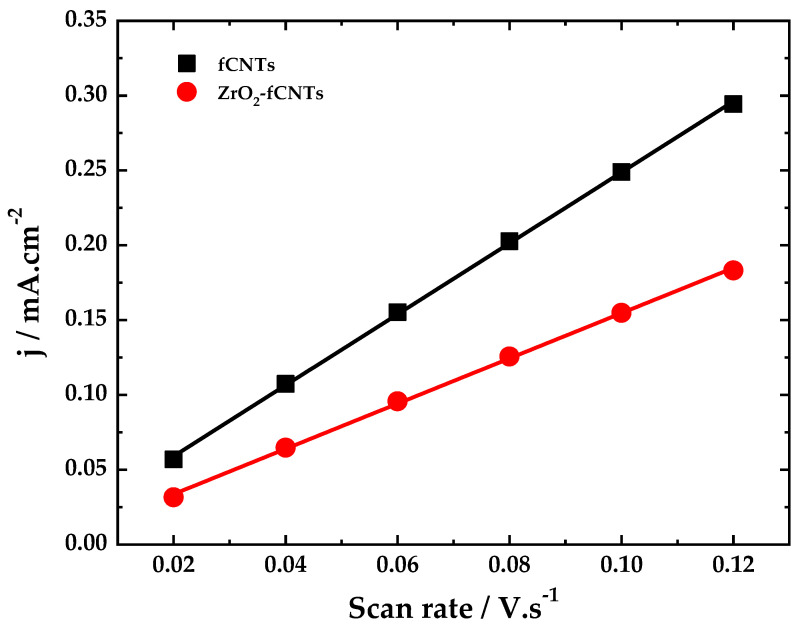
Linear regression of background current density respect to the scan rate (capacitance).

Electrochemical capacitance, C_dl,_ of the modified electrodes was studied by CV, this was carried out in 0.1 mol·L^−1^ PBS (pH 6.8) and potentials from −0.75 V to −0.2 V [52]. For the CV and according to Figure 8, the background current is a function of the scan rate, and is described by the following Equation (2):

j = I_c_/A = C_dl_ ν (2)

where the A is the effective surface area, ν is the scan rate, and C_dl_ is the double layer capacitance. The plot of current density (I_c_/A) versus scan rate (ν) gave a straight line where the slope is (C_dl_) of the electrode. Figure 9 shows the linear regression of background current (charging current) respect to the scan rate. The C_dl_ obtained for PB/fCNTs/GC was 2.37 mF·cm^−2^, and the C_dl_ of the PB/ZrO_2_-fCNTs/GC electrode was 1.51 mF·cm^−2^. This indicates that PB/ZrO_2_-fCNTs/GC electrode exhibited lower electrode-specific capacitance than PB/fCNTs/GC electrode. This result effectively confirms the results obtained by BET surface area.

### 3.9. Stability of PB Films

Haghighi et al. [54] reported that PB films showed decay due to both non-faradaic (presence of OH^−^ at high pH buffers) as well as faradaic processes (${\mathrm{Fe}}_{4}^{\mathrm{III}}{\left[{\mathrm{Fe}}^{\mathrm{II}}{\left(\mathrm{CN}\right)}_{6}\right]}_{3}\left(\mathrm{PB}\right)+12{\mathrm{OH}}^{-}\to 4\mathrm{Fe}{\left(\mathrm{OH}\right)}_{3}+3{\mathrm{Fe}}^{\mathrm{II}}{\left(\mathrm{CN}\right)}_{6}^{4-}$). Where the OH^−^ in formation of ferric hydroxide is responsible for the cleavage of the PB. The PB modified electrodes were quite stable in 0.1 mol·L^−1^ PBS (pH 6.8) solutions. Peak current did not decrease through cycling from 10 to 100 times at a sweep rate of 50 mV (not shown). In agreement to the studies of zeta potential (Figure 5) both fCNTs and ZrO_2_-fCNTs composites have surface negative charge. We have associated these results to the capacity of the composites to avoid the formation of superficial OH^−^ that originate decay off the PB film.

The reproducibility of the modified electrodes was performed by testing ten electrodes, manufactured in the same way, all of them showed an acceptable reproducibility with a RSD average of 2.0–3.6%. The long-term storage stability of the modified electrodes was examined using procedures already reported [55]. After a 30-day storage period, the PB/ZrO_2_-fCNTs/GC modified electrode still retained 95% of its initial current response which indicated that it had a good stability, while the PB/fCNTs/GC electrode only retained 80% of its initial current response.

### 3.10. Cyclic Voltammetry Behavior of the PB/ZrO_2_-fCNTs/GC Modified Electrodes in Presence of Hydrogen Peroxide

**Figure 10:**
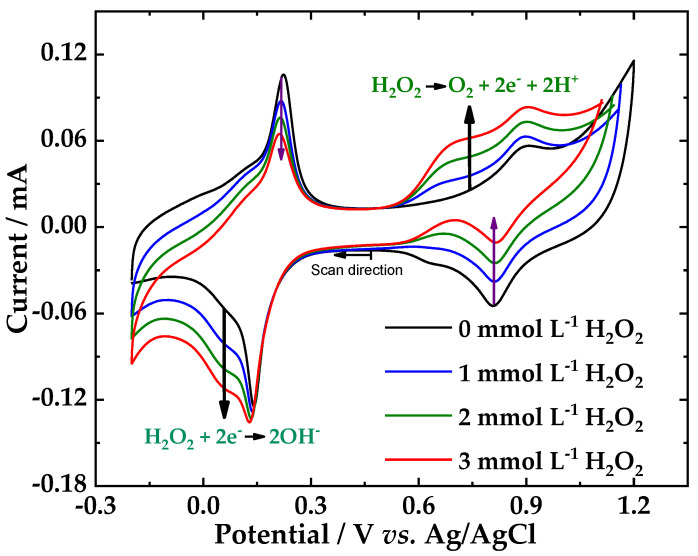
Cyclic voltammograms of hydrogen peroxide at PB/ZrO_2_-fCNTs/GC electrode in a 0.1 mol·L^−1^ PBS (pH 6.8) solution. Scan rate 40 mV·s^−1^.

On conventional electrodes, peroxide reactions require high overvoltage, either reduction or oxidation, reactions 3 and 4 [55]. The challenge in the literature is to produce chemically modified electrodes, at which the overvoltage, both the electrochemical H_2_O_2_ oxidation and reduction reaction can be reduced so that measurements can be performed at oxidation potentials less than +1.0 V and reduction potentials below −0.1 V (*vs.* SCE) [54], respectively. According to our previous results [47], the PB/fCNTs/GC electrode exhibited an unstable behavior at the potentials applied for detection H_2_O_2_. Therefore, the PB/ZrO_2_-fCNT/GC modified electrode was considered the best electrode for the evaluation H_2_O_2_ detection in terms of operability. Figure 10 shows cyclic voltammograms of hydrogen peroxide at PB/ZrO_2_-fCNTs/GC electrode, where well-defined redox peaks were obtained. In the presence of H_2_O_2_, both zones of the voltammogram showed a marked decrease in the reverse redox currents and both zones showed an increase in the forward redox currents, demonstrating that the electrocatalytic reduction and oxidation of hydrogen H_2_O_2_ occurred in both zones. The redox peaks at +0.6 V presented an electrocatalysis towards the oxidation of H_2_O_2_ (reactions 4), while the redox peaks at +0.2 V electrocatalyzed the H_2_O_2_ reduction (reactions 3). PB/ZrO_2_-fCNTs/GC electrode shows a decrease in the over-voltages to reduce or oxidize peroxide. Figure 10 also demonstrates that at PB/ZrO_2_-fCNTs/GC electrode the electroxidation of hydrogen peroxide is more effective, due to the better response of the modified electrode to increased peroxide concentration. In addition, the anodic peak current increased by ca. 25 and 10% and the cathodic peak decreased by ca. 20 and 30%, clearly indicating a catalytic oxidation reaction.

Reduction reactions

(R3) $$K_{4}{\mathrm{Fe}}_{4}^{\mathrm{II}}{\left[{\mathrm{Fe}}^{\mathrm{II}}{\left(\mathrm{CN}\right)}_{6}\right]}_{3}+2H_{2}O_{2}⇄{\mathrm{Fe}}_{4}^{\mathrm{III}}{\left[{\mathrm{Fe}}^{\mathrm{II}}{\left(\mathrm{CN}\right)}_{6}\right]}_{3}+4{\mathrm{OH}}^{-}+4K^{+}$$

Oxidation Reactions

(R4) $$2{\mathrm{Fe}}_{4}^{\mathrm{III}}{\left[{\mathrm{Fe}}^{\mathrm{III}}{\left(\mathrm{CN}\right)}_{6}\right]}_{3}A_{3}+3H_{2}O_{2}⇄2{\mathrm{Fe}}_{4}^{\mathrm{III}}{\left[{\mathrm{Fe}}^{\mathrm{II}}{\left(\mathrm{CN}\right)}_{6}\right]}_{3}+3O_{2}+6A^{-}+6H^{+}$$

### 3.11. Electrochemical Detection of H_2_O_2_ at PB/ZrO_2_-fCNTs/GC Electrode

**Figure 11:**
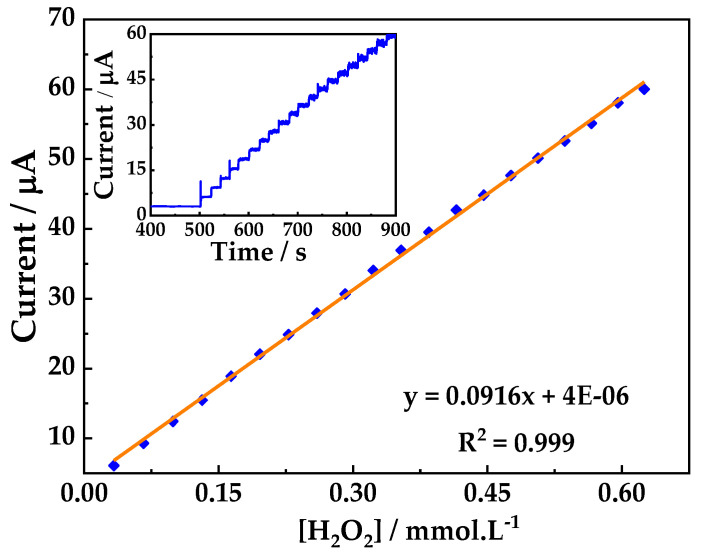
Chronoamperometric detection of H_2_O_2_ at the PB/ZrO_2_-fCNTs/GC electrode, in a 0.1 mol·L^−1^ PBS (pH 6.8) solution and its calibration curve. Working potential of 1.0 V vs. Ag/AgCl.

The electrocatalytic oxidation of H_2_O_2_ at PB/ZrO_2_-fCNTs/GC modified electrode was studied using CA (Figure 11). The current signal was linear for H_2_O_2_ concentrations (range of 3 × 10^−5^ to 6 × 10^−4^ mol·L^−1^, y = 0.0916x + 4 × 10^−6^, R^2^ = 0.999). Quantification limit (LQ) was 10.91 μmol·L^−1^ and detection limit (LD) of 3.5913 μmol·L^−1^. Due to these results, we suggest the PB/ZrO_2_-fCNTs/GC modified electrode to detect peroxide in the oxidation zone.

Current signals at PB/ZrO_2_-CNTs/GC in the presence of H_2_O_2_, ($I_{c}$), and in the absence of H_2_O_2_, ($I_{L}$), were used to evaluate the rate constant, $K_{c},$ for the catalytic reaction, Equation (3) [56]:

(3) $$\frac{I_{c}}{I_{L}}={\left(K_{c}C\pi \right)}^{1/2}t^{1/2}$$

where $K_{c}$, C, and t are the rate constant of the catalytic chemical reaction (cm^3^ mol^−1^ s^−1^), the bulk concentration of H_2_O_2_ (mol·cm^−3^) and the fixed time (s), respectively. From the plot of $I_{c}/I_{L}$ versus $t^{1/2}$ slope (not shown), the value of $K_{c}$ was obtained for a fixed concentration of H_2_O_2_. The $K_{c}$ mean value in the concentration range 3 × 10^−5^ to 6 × 10^−4^ mol·L^−1^ was 10.73 × 10^6^ cm^3^·mol^−1^·s^−1^, which is about 3.5 times lower than the value of 3 × 10^6^ cm^3^·mol^−1^·s^−1^ reported for PB [57,58].

### 3.12. Comparison of Results

Table 6 shows the comparison of the results obtained, in Section 3.11, with others previously reported in the literature. PB/ZrO_2_-fCNTs/GC modified electrode has better detection limit than other reports, suggesting that the electrode coating can be used successfully to sense H_2_O_2_.

## 4. Conclusions

It was proved that the fCNTs and ZrO_2_-fCNT improved the electron transfer behavior of the GC electrode. The changed surface area of fCNTs by in situ zirconia nanoparticles synthesis induces changes in the electron transfer behavior. Furthermore, the fCNTs and ZrO_2_-fCNTs layers exhibit good compatibility and affinity to the PB layer. The applicability of the sensor for detection of hydrogen peroxide at the ZrO_2_-fCNTs/GC electrode was demonstrated. The best electrochemical detection and linear range detection is shown by the ZrO_2_-fCNTs/GC modified electrode. Based on these advantages, the fabricated sensor exhibits good electrochemical sensibility, reversibility, and excellent linear relationship, nonetheless, the detection limit of the electrode can be improved, especially enhancing the conditions of hydrogen peroxide detection. Finally, the zirconia doped carbon nanotubes can be used in the development of enzyme based biosensors, which is the basis for future studies in our laboratory.

## Figures and Tables

**Table 1:** Specific surface area and pore volume of fCNTs, ZrO_2_-fCNTs and ZrO_2_ nanoparticles.

Sample	Area BET (m^2^·g^−1^)	BJH Pore Volume (cm^3^·g^−1^)
fCNTs	298.4	2.52
ZrO_2_-fCNTs	92.12	0.20
ZrO_2_	249.1	---

**Table 2:** Thermal analysis of pCNTs, fCNTs and ZrO_2_-fCNTs.

Sample	First Stage		Weight Loss (%)	Second Stage		Weight Loss (%)	Percentage of ZrO_2_
	Ti (°C)	T Decomp. (°C)		Ti (°C)	T Decomp. (°C)		
pCNTs	426	724	91	---	---	---	---
fCNTs	504	707	97	---	---	---	---
ZrO_2_-fCNTs	80	150	10	---	---	---	40
	276	347	2	366	597	40	

**Table 3:** XRD parameters of CNT samples of the (002) plane.

CNT Sample	2θ (°)	d (Å)	FWHM (°)	Intensity (a.u)
Pristine	25.36	3.59	3.96	384
Functionalized	25.18	3.61	3.73	885

**Table 4:** Diffraction angle values of ZrO_2_-fCNTs and ZrO_2_, and their miller indexes.

Reflection Angle (2θ) (°)		Miller Index
ZrO_2_-fCNTs	ZrO_2_ Cubic Phase	
30.30	30.51	(111)
35.25	35.19	(200)
50.54	50.68	(220)
60.24	60.33	(311)
63.08	63.21	(222)
74.62	74.74	(400)

**Table 5:** Diameter distribution of CNT samples.

CNT Sample	Diameter (nm)	Distribution of Diameter (nm)
Pristine	18 ± 4	12–25
Functionalized	12 ± 2	7–16

**Table 6:** Comparison of the results obtained at modified electrodes with other electrode reported in the literature.

Modified Electrode	Sensitivity (µA mM^−1^)	Detection Limit (mM)	Detection Potential (V)	Ref.
PB/ZrO_2_-fCNTs/GC	91.3	0.00359	+1.0	this work
PB-fCNTs/GC.	163.01	0.015	0.00	[47]
HRP-TiO_2_/fCNTs/GC	963	0.81	−1.50	[47]
HRP from leaves of Guinea grass/graphene.	39.93	0.15.	−0.65	[59]
CuInS_2_-graphene/HRP.	11.2	0.047	−0.2	[60]
Prussian blue nanocubes on reduced graphene oxide.	Not reported	0.04	0.2	[61]
HRP/chitosan-gelatin composite biopolymers nanofibers/graphite electrode.	44	0.05	−0.30	[62]
Pt/Au	22.181	0.06	−0.20	[63]

## Abbreviations

- Prussian blue (PB)
- glassy carbon (GC)
- zirconia (ZrO_2_)
- carbon nanotubes (CNTs)
- pristine carbon nanotubes (pCNTs)
- functionalized carbon nanotubes (fCNTs)
- Berlin green (BG)
- Prussian white (PW)
- poly(diallyldimethylammonium chloride) (PDDA)
- cyclic voltammetry (CV)
- chronoamperometry (CA)
- anion supplied by the electrolyte (A^-^)
- quantification limit (LQ)
- detection limit (LD)
- thermogravimetric analysis (TGA)
- transmission electron microscopy (TEM)
- field emission scanning electron microscopy (FESEM)
- atomic force microscopy (AFM)
- X-ray diffraction (XRD)
- Fourier transform infrared (FTIR)
- dimethylformamide (DMF)
- Brunauer–Emmett–Teller (BET)
- Barrett–Joyner–Halenda (BJH)
- multi-walled carbon nanotubes (MWCNTs)
- International Union of Pure and Applied Chemistry (IUPAC)
- horseradish peroxidase (HRP)
- surface concentration ($\Gamma_{C}$)
- geometric area (A)
- current (I)
- current density (j)
- anodic peak current (I_pa_)
- cathodic peak current (I_pc_)
- half-wave potential (E_1/2_); peak-to-peak separation potential (∆E)
- peak current (I_p_)
- number of electrons involved in the redox process (n)
- F = 96 485 C mol(e)^−1^
- Scan rate (ν)
- R = 8.314 J K^−1^ mol^−1^ and temperature (T)
- rate constant for the catalytic reaction ($K_{c}$)
- time (t)
- double layer capacitance (C_dl_).
